# Supplementary material for: Evaluation of Pollution Level, Spatial Distribution, and Ecological Effects of Antimony in Soils of Mining Areas: A Review
Source: Int J Environ Res Public Health. 2022 Dec 23;20(1):242. doi: 10.3390/ijerph20010242 (PMC9819699; doi:10.3390/ijerph20010242)
Supplement: Supplementary file 1 [file ijerph-20-00242-s001.zip › ijerph-2034432-supplementary.pdf]

Supplementary Material

Pages = 10

Figures=2

Tables =2

Table S1. Search logic and inclusion-exclusion criteria used for the systematic literature search and database creation.

Article identification strategy
**Search logic**
Antimony/Sb, soil, mining
**Inclusion criteria**
Reference is written in English
Published date between the years 1989-2021
Soil Sb concentrations are specified
Geographical location of study area is clearly provided
Research in soil Sb pollution related to mining and smelting activities
The sampling and processing methods used are all widely accepted by the scientific community
**Exclusion criteria**
Unrelated research field
Only concentration range or in the form of figure of soil Sb are presented
Soil Sb concentrations are not clearly provided including review articles

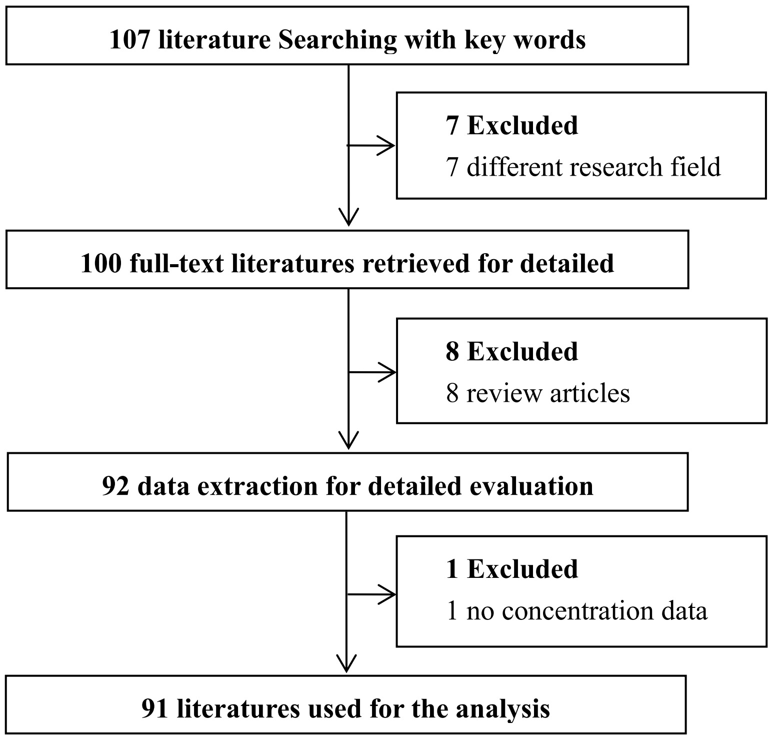

Figure S1. Literature selection flow chart.

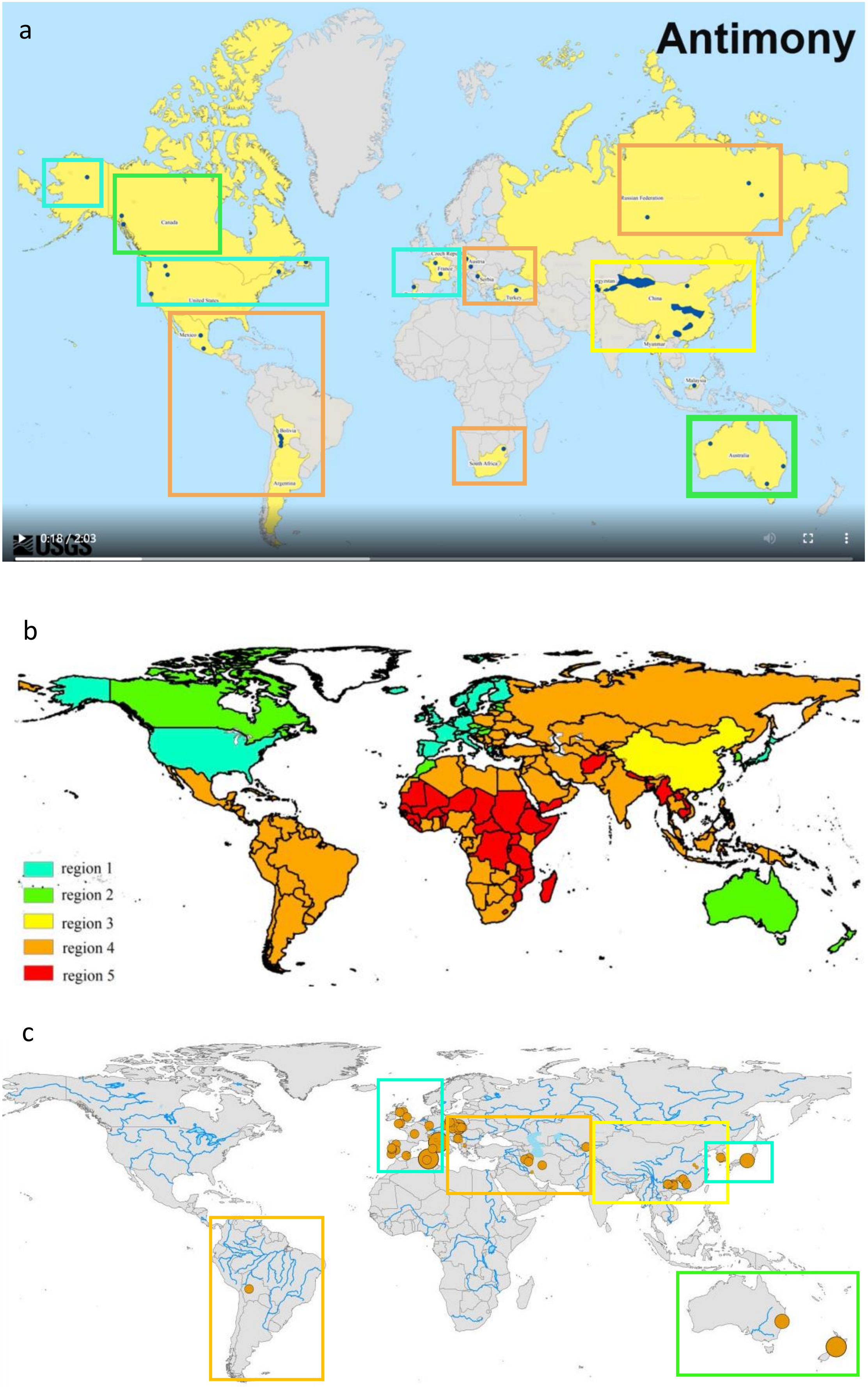

Figure S2. (a) Global distributions of Sb resources (Labay et al., 2017). (b) Spatial

distributions for five regions in the world with different levels of economic development and emission control technologies (Zhu et al., 2020). (c) Spatial distribution of Sb concentrations in soils in different regions based on existing data. Four color boxes of (c) correspond to the color of regions in (b).

Conditional inference tree (CIT) model

CITs can be used to establish a model of the regression relationship between explanatory variables (X) and response variables (Y) (Hothorn et al., 2006). CIT procedure assumes that the conditional distribution D (Y|X) of the response variable Y is a function that depends on of the covariates X.

$$D\ (Y|X)=D\ (Y|X_1,\ X_2,\ \ldots,\ X_m)=D\ (Y|f\,(X_1,\ X_2,\ \ldots,\ X_m))$$

$$Ln = \{(Y_i,\ X_i);\ i = 1,\ \ldots,\ n\}$$

For a given learning sample Ln, the recursive binary partition can be expressed as a non-negative integer value weight. When the corresponding observed value is the element of the node, each tree node is represented by the case weight vector of the non-zero element, otherwise, it is zero. CIT uses a chi-square test to overcome problems such as over-fitting and selection bias for continuous variables or variables with many categories.

The general partitioning procedure for the construction of the CIT involves three steps. First, the X_i showing the strongest relation with Y is identified. For the given case weight *w*, statistical significance tests are used to obtain the relationship between Y and m-dimensional covariates X. The global hypothesis of independence is formulated as $H_0 = \bigcap_{i=1}^{m} H_0^i$, and the partial hypothesis H_0^i is defined as H_0^i : D(Y|X_i)= D(Y). Partitioning is stopped if the global hypotheses cannot be defeated under pre-specified

condition. Otherwise, the covariate with the strongest relationship to Y is selected for further splits of the node. Second, the selected covariate X_{i*} is used to find the optimum binary split. For each possible splitting, two-sample linear statistics are conducted to assess the goodness of the partitioning by finding the discrepancy between the two subgroups with no intersection $\{Y_i \mid w_i > 0 \wedge X_i \in A; i=1, \ldots, n\}$ and $\{Y_i \mid w_i > 0 \wedge X_i \notin A; i=1, \ldots, n\}$. The two case weights w_{left} and w_{right} are determined according to the results of the statistics. Third, the above-mentioned two-stage process is repeated with updated case weights w_{left} and w_{right}.

The output result of the CIT method is a tree-structured recursive partitioning similar to a flow chart. The topmost node of the tree is the root node. Each internal node represents a test on a property, and each branch corresponds to an output of the test. Each external node at the bottom represents a determined category. The path from the root node to any external node can be easily converted into a simple rule, such as "if-then". Decision trees can be binary or multipartite. It is generally believed that binary trees are more accurate than traditional trees and form fewer data fragments. Highly leveraged data are detected and removed by Mahalanobis distance, univariate analyses, and pruning algorithms to develop powerful decision trees.

In this study, a CIT was developed for the concentrations of Sb based on seven specific predictors: pH (pH), organic matter (OM), grain-size distribution (sand, silt, clay,) electrical conductivity (EC) of soil.

Non-carcinogenic risks assessment method

Non-carcinogenic risk assessment (adult and child) was calculated using Eqs. (1) (US EPA, 1989):

$$ADD_{ing} = \frac{C \cdot IngR \cdot EF \cdot ED}{BW \cdot AT} \times 10^{-6} \qquad \text{(S1)}$$

where ADD_{ing} is the average daily intake dose of heavy metals via oral ingestion of soil, water and food (mg/kg/day), C is the heavy metal concentration found in the soil (mg/kg), $IngR$ is the ingestion rate of soil (mg/day). EF is the exposure frequency (day/year), ED is the exposure duration (year), BW is the body weight of the exposed individual (kg), AT is the time period over which the dose is averaged (day).

Non-carcinogenic hazards are typically characterized by the hazard quotient (HQ) and were calculated using Eqs. (2) (US EPA, 1989):

$$HQ = \sum HQ_{\mathrm{i}} = \sum \frac{ADD_{ij}}{RfD_{ij}} \qquad \text{(S2)}$$

where RfD is respective reference dose (mg/kg-day). The value of $\mathrm{RfD_{ing}}$ for Sb is 0.0004.

Table S2. Exposure factors for health risk assessment models.

Factor	Definition	Unit	Value	
			Children	Adults
AT	Average life span for heavy metals	d	70×365	70×365
BW	Average body weight	kg	19	62
ED	Exposure duration	year	6	25
EF	Exposure frequency	d/a	350	350
IngR	Ingestion rate	mg/d	24	25

References

Hothorn, T., Hornik, K., Zeileisa, A., 2006. Unbiased recursive partitioning: A conditional inference framework. J. Comput. Graph. Stat. 15 (3), 651-674.

US EPA 1989. Risk assessment guidance for Superfund. Human health evaluation manual, (part A) [R], vol. 1. Washington, DC: Office of emergency and remedial response. [EPA/540/1-89/002].

Zhu, C.Y., Tian, H.Z., Hao, J.M., 2020. Global anthropogenic atmospheric emission inventory of twelve typical hazardous trace elements, 1995-2012. Atmos. Environ. 220, 117061.
